# Parenchyma-sparing resection for a complex jejunal duplication with a shared mesenteric blood supply: a case report

**DOI:** 10.1093/jscr/rjaf871

**Published:** 2025-11-06

**Authors:** Abuzar Farhad, Fazeela Bibi, Khalil EL ABDI, Aleena Amir Malik, Suraksha Rani, Ali Sher, Kristen Batten, Azwa Zubair, Kanza Ahmed, Said Hamid Sadat

**Affiliations:** Department of Surgery, Khyber Teaching Hospital, University Road, Rahatabad, Peshawar 25120, Pakistan; Jinnah Medical and Dental College, Shaheed-e-Millat Road, Karachi East District, Karachi Sindh 74800, Pakistan; Faculty of Medicine and Pharmacy of Rabat, Mohammed V university, Avenue Mohammed Belarbi El Alaoui, BP 6203, Rabat Institutions, Souissi, Rabat 10100, Morocco; Department of Surgery, United Medical and Dental College, UMDC Road Near Nasir Jump Bus Stop, Sector 48 H Korangi Creek, Karachi, Sindh 25120, Pakistan; Department of Medicine, Jinnah Medical and Dental College, Shaheed-e-Millat Road, Karachi East District, Karachi Sindh 74800, Pakistan; Rahbar Medical and Dental College, affiliated with the University of Health Sciences, Harbanspura Road, Lahore Cantt, Lahore, Pakistan; Saint James School of Medicine, The Quarter, The Valley, Anguillz, United Kingdom; Department of Medicine, Baqai Medical University, 51, Deh Tor, Gadap Road, PO Box No 2407, Karachi-74600, Pakistan; Department of Surgery, United Medical and Dental College, UMDC Road Near Nasir Jump Bus Stop, Sector 48 H Korangi Creek, Karachi, Sindh 25120, Pakistan; Kabul University of Medical Sciences Abu Ali Ibn Sina, University Road Kabul 1001, Afghanistan

**Keywords:** jejunal duplication cyst, parenchyma-sparing resection, pediatric surgery, bowel obstruction, surveillance

## Abstract

Gastrointestinal (GI) duplication cysts present significant diagnostic and surgical challenges, particularly with complex anatomy. We report a 2-year-old male with chronic obstructive symptoms caused by a mixed-type jejunal duplication. While initial ultrasound was equivocal, contrast-enhanced computed tomography delineated a cystic mass on the mesenteric border, guiding surgical planning. Intraoperatively, a shared mesenteric blood supply with the native jejunum precluded complete resection. A parenchyma-sparing partial excision of the symptomatic, non-communicating portion was performed, preserving the fused communicating segment. Histology confirmed the diagnosis. The patient’s recovery was uneventful, with complete symptom resolution at follow-up. This case illustrates that for complex GI duplications, a tailored, parenchyma-sparing resection is a safe and effective strategy when vascular integrity is at risk, shifting the clinical focus from acute management to the necessity of long-term surveillance for the retained segment.

## Introduction

Gastrointestinal (GI) duplication cysts are rare congenital anomalies, occurring in ~1 in 4500 live births [1]. Their cardinal features include an intimate attachment to the alimentary tract, a shared smooth muscle wall, and, critically, a common mesenteric blood supply with the adjacent native bowel [1, 2]. The established standard of care is complete surgical resection, which is curative and prevents future complications such as obstruction, bleeding, or malignant transformation [3].

However, this therapeutic principle is often challenged by complex anatomy. The shared vasculature between the duplication and the native intestine creates a critical surgical dilemma: aggressive en bloc resection risks ischemic injury to the healthy bowel, while incomplete removal may leave a source for future complications [3]. In such scenarios, surgeons must employ parenchyma-sparing techniques, such as partial excision or mucosal stripping, to preserve bowel viability [4].

While surgically prudent, this necessary compromise shifts the clinical focus from an acute, curative intervention to a chronic management problem. The retained segment, although often asymptomatic, carries a recognized long-term risk of malignant transformation, with adenocarcinoma being the most reported histology [5]. Despite this risk, no consensus guidelines exist for the surveillance of retained duplication segments, representing a critical knowledge gap in the field [6].

Herein, we present the case of a two-year-old boy with a complex, mixed-type jejunal duplication cyst managed with a parenchyma-sparing resection. This case serves to highlight the transition from an acute surgical problem to a chronic surveillance dilemma and underscores the urgent need for evidence-based follow-up strategies.

### Patient presentation and initial workup

A 2-year-old male presented with a chronic history of intermittent, non-progressive abdominal distention and bilious vomiting since infancy. He had two previous hospital admissions for these symptoms, which were managed conservatively with intravenous fluids, antibiotics, and nasogastric decompression, providing only transient relief.

On physical examination during the current admission, the patient was afebrile and hemodynamically stable, with anthropometric measurements appropriate for his age. The abdomen was visibly distended and tympanitic to percussion, with mildly exaggerated bowel sounds. There was no palpable mass, organomegaly, tenderness, or peritoneal signs. Laboratory investigations revealed a mild leukocytosis (11.3 × 10^9^/L), thrombocytosis (524 × 10^9^/L), and a microcytic hypochromic anemia (hemoglobin: 11.3 g/dl). Serum electrolytes, renal and liver function tests, and C-reactive protein were within normal limits.

### Diagnostic imaging

**Figure 1:**
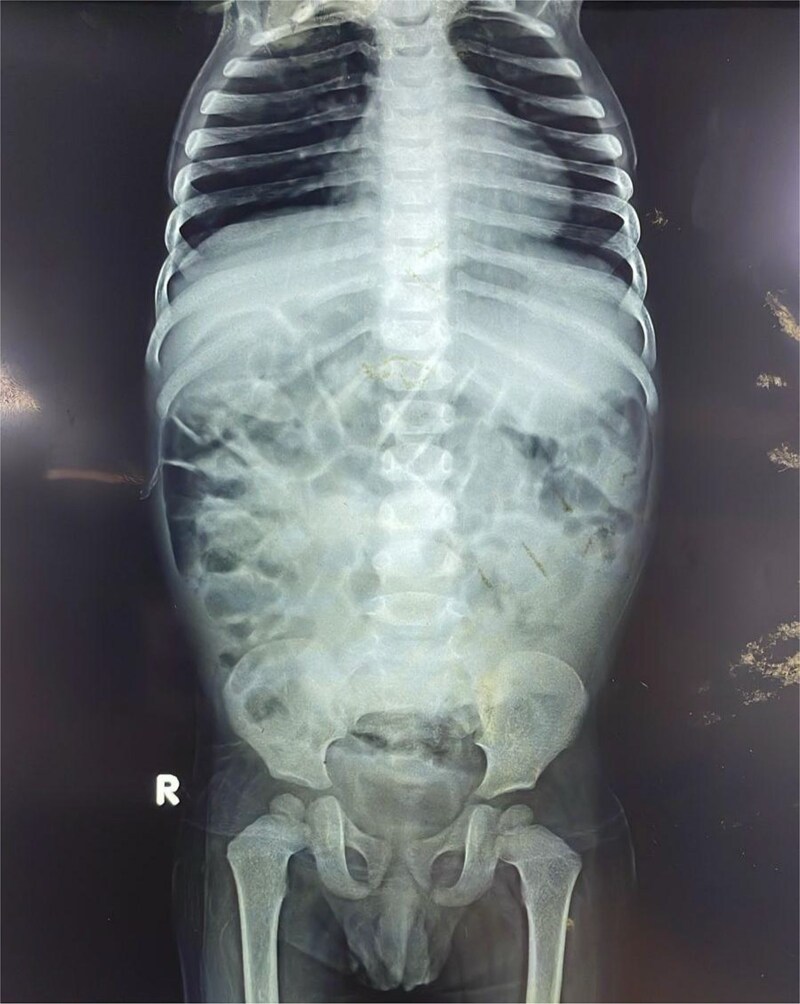
Anteroposterior abdominal radiograph demonstrating diffusely air-distended small bowel loops consistent with a partial small bowel obstruction. No significant air-fluid levels or evidence of pneumoperitoneum is visible.

Initial plain radiographs of the abdomen and chest (Fig. 1) revealed diffusely air-distended bowel loops but no air-fluid levels or pneumoperitoneum, suggesting a partial obstructive process. An abdominal ultrasound performed during a prior admission had identified a 6.0 × 5.0 cm thick-walled, fluid-filled gut segment in the right iliac fossa, provisionally diagnosed as a Meckel’s diverticulum.

**Figure 2:**
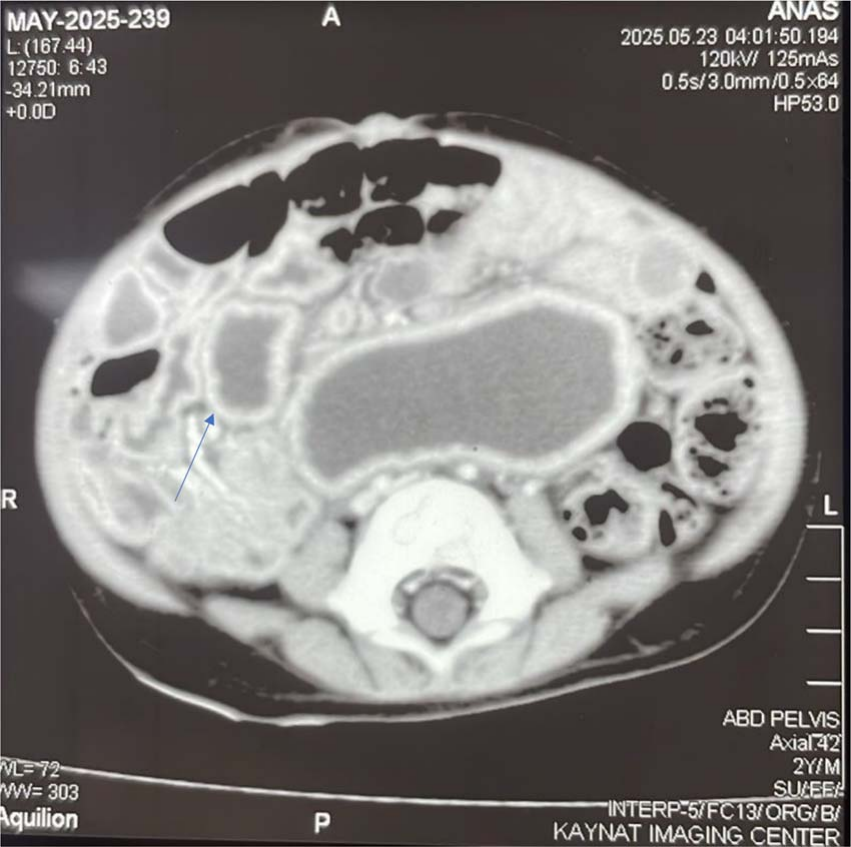
Contrast-enhanced axial CT scan of the abdomen. The image reveals a well-defined, thick-walled cystic lesion (arrow) located along the mesenteric border of the midgut. The lesion demonstrates peripheral post-contrast enhancement without internal septations or solid components, findings highly suggestive of a jejunal duplication cyst.

To better characterize this lesion, a contrast-enhanced computed tomography (CT) scan of the abdomen and pelvis was performed. The scan (Fig. 2) revealed a well-defined, thick-walled cystic lesion in the midgut region that demonstrated peripheral post-contrast enhancement without internal septations or solid enhancing components. Its intimate location along the mesenteric border of the small bowel was highly suggestive of an enteric duplication cyst.

### Surgical intervention and operative findings

Given the persistent obstructive symptoms and definitive imaging findings, the patient underwent an elective exploratory laparotomy. The exploration confirmed a complex jejunal duplication cyst originating from the mid-jejunum. The cyst had two distinct components: a proximal, dilated, blind-ending, and non-communicating segment that was causing intermittent obstruction; and a distal segment that was fused with the native bowel wall and communicated with the intestinal lumen (Video 1).

**Figure 3:**
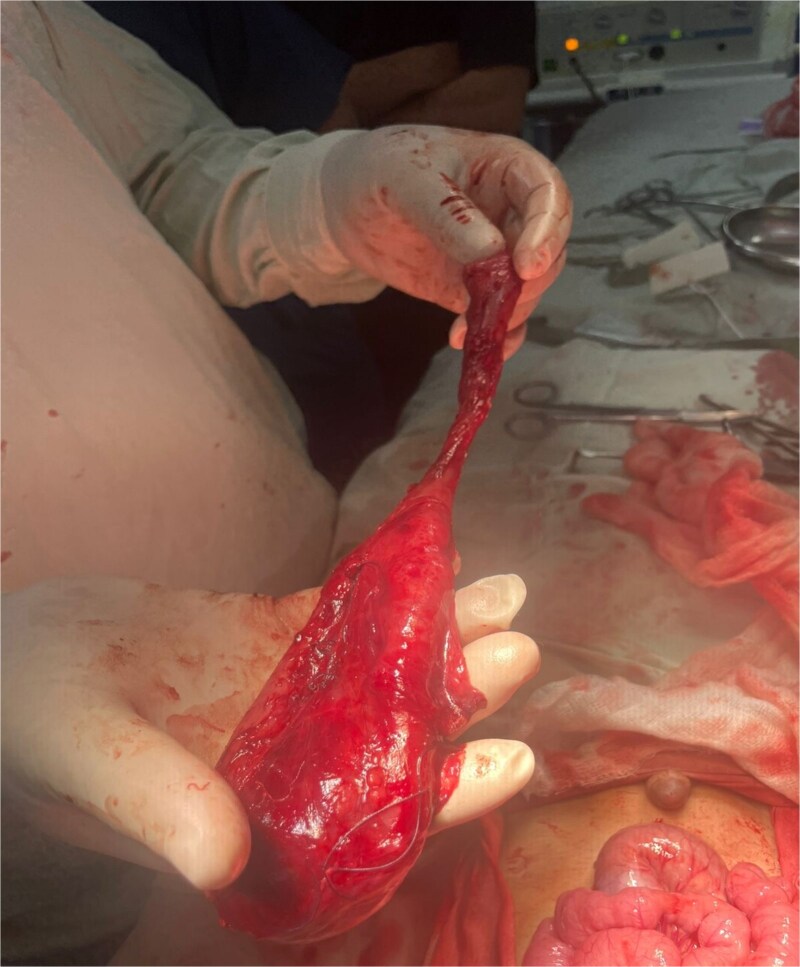
Intraoperative picture showing the removed non-communicating proximal cyst.

Crucially, the distal communicating portion shared a common mesenteric blood supply with the adjacent jejunum. Complete excision of the entire duplication was therefore deemed to carry an unacceptably high risk of inducing ischemia in the native bowel. The surgical strategy was consequently tailored to the anatomy: the symptomatic, non-communicating proximal cyst was resected (Fig. 3), while the asymptomatic, fused distal segment was left in situ after its communication point was ligated.

### Postoperative outcome and follow-up

The patient’s postoperative course was uneventful. Oral intake was initiated on the first postoperative day, and he was discharged on the third day. At a 2-week follow-up appointment, the patient had recovered fully and remained asymptomatic, with complete resolution of his vomiting and abdominal distention. At a subsequent two-month follow-up, he was thriving, showing appropriate weight gain and reporting normal bowel function.

## Discussion

This case of a mixed-type jejunal duplication cyst exemplifies the diagnostic and therapeutic challenges inherent to this condition. The patient’s protracted history of non-specific obstructive symptoms is characteristic, often leading to diagnostic delays due to clinical mimicry of more common pediatric conditions. The initial provisional diagnosis of a Meckel’s diverticulum on ultrasound is a common pitfall. However, the subsequent CT finding of a mesenteric-border jejunal lesion argued strongly against this, as Meckel’s diverticula are typically located on the anti-mesenteric border of the distal ileum [7]. While a mesenteric cyst remained a possibility, the mass’s intimate association with the bowel wall was the key radiological feature favoring a duplication cyst [6].

The operative findings presented a classic conflict between two core surgical tenets: the principle of complete resection to prevent symptom recurrence and future complications [1, 3], versus the functional imperative of preserving bowel viability. A complete *en bloc* resection of the duplicated segment, while theoretically ideal, would have unacceptably compromised the mesenteric blood supply. In such scenarios, a parenchyma-sparing approach is not merely an alternative but the standard of care [4].

While surgically pragmatic, the decision to leave a portion of the duplicated segment in situ shifts the clinical focus from an acute, curative intervention to a chronic management problem. The most significant long-term risk is malignant transformation within the residual mucosa, with adenocarcinoma being the most reported histology [5]. This potential transforms an anatomical anomaly into a lifelong clinical concern that mandates structured surveillance. This necessity exposes a critical knowledge gap: currently, no consensus guidelines exist for the surveillance of retained, asymptomatic duplication segments [6]. This case transitions the focus from a solved surgical problem to an unsolved surveillance dilemma, underscoring the urgent need for evidence-based follow-up protocols.

While acknowledging the current lack of formal guidelines, a practical surveillance protocol can be proposed to manage the long-term risk of malignant transformation [8]. Such a plan should involve a multimodal approach, beginning with regular clinical follow-up to monitor for any new or recurrent symptoms like pain, obstruction, or weight loss [9]. This should be complemented by periodic imaging. Abdominal ultrasound is an effective and non-invasive first-line modality, frequently used for both prenatal and postoperative monitoring of duplication cysts [9]. Based on protocols for other postoperative scenarios, a reasonable schedule could involve an initial ultrasound at 6 to 12 months after surgery, followed by evaluations every 1 to 2 years if the patient remains asymptomatic and the retained segment is stable [10]. For retained segments that are endoscopically accessible, periodic endoscopic evaluation, with or without endoscopic ultrasound, could offer direct mucosal visualization and the opportunity for biopsy, which is particularly relevant given that adenocarcinoma is the most reported histology in these cases [11]. This proactive, structured follow-up balances the low but significant risk of malignancy against the burden of intervention, providing a safeguard for the patient until evidence-based consensus guidelines are established.

## Conclusion

This case of a mixed-type jejunal duplication underscores that individualized, anatomy-driven surgical planning is paramount, especially when a shared mesenteric blood supply precludes complete resection. Our successful partial excision of the symptomatic, non-communicating segment validates a parenchyma-sparing approach, which effectively resolves obstructive symptoms while mitigating the risk of iatrogenic intestinal ischemia. Although this strategy is surgically prudent, it leaves a communicating segment in situ, creating a long-term clinical imperative for surveillance to monitor for rare but significant complications, such as malignant transformation. While limited by its single-case nature, this report reinforces that for complex GI duplications, preserving bowel viability is a justifiable priority over complete excision, provided a robust follow-up plan is implemented. We propose a practical surveillance protocol centered on periodic clinical review and ultrasound, which serves as a pragmatic approach for monitoring retained segments until formal consensus guidelines are established.

## Supplementary Material

video_1_rjaf871
